# The inCASA project: improving the quality of life and social care for the ageing population

**Published:** 2012-06-15

**Authors:** Andreas P Kapsalis, Georgios Lamprinakos, Konstantinos A Papadopoulos, Dimitra I Kaklamani, Iakovos S Venieris

**Affiliations:** School of Electrical and Computer Engineering, National Techical University of Athens, Heroon Polytechniou 9, GR-15773, Athens, Greece; School of Electrical and Computer Engineering, National Techical University of Athens, Heroon Polytechniou 9, GR-15773, Athens, Greece; School of Electrical and Computer Engineering, National Techical University of Athens, Heroon Polytechniou 9, GR-15773, Athens, Greece; School of Electrical and Computer Engineering, National Techical University of Athens, Heroon Polytechniou 9, GR-15773, Athens, Greece; School of Electrical and Computer Engineering, National Techical University of Athens, Heroon Polytechniou 9, GR-15773, Athens, Greece

**Keywords:** telehealth, telecare, service-oriented architecture, habits profiling, assisted-living

## Abstract

This paper describes an ICT platform aiming to support the well-being of frail elderly people and facilitate them to stay longer and more healthily in their own home. Its principal characteristic is the combination of Telehealth and Telecare monitoring in a unified way, allowing the simultaneous health, mental and psychological status evaluation of an elderly person. For this purpose the platform enables the deployment of services to follow-up the patient’s health status based on a set of monitored parameters per disease, to track the suitability of the in-house environmental conditions and finally to profile user’s habits and diagnose deviations from their usual activities.

The inCASA project implements such platform based on a Service-Oriented Architecture which relies on the Hydra Middleware. Hydra is receiving measurements from proprietary Telehealth and Telecare gateways deployed in the home premises and transforms them into Health Level 7 (HL7) compliant data. Platform developers may add business logic and create healthcare applications on top of the Middleware without getting involved with low-level communication issues with the various types of sensor devices and their protocols. Another core module of the architecture is the Smart Personal Platform (SPP) in which the patient data are forwarded from Hydra, stored and analyzed. SPP includes a reasoning mechanism responsible for the comparison of retrieved measurements with specified thresholds per monitored parameter and per patient. Furthermore, this mechanism detects deviations from the stored habits profile of each user which is dynamically built based on history data. Either in the case of thresholds exceeding or in the case of habits profile deviation, alerts are generated and classified based on their severity. Both data and alerts are available in the back-end user interface of the platform, the so-called Consumer Application interface which is the single point of access for the inCASA operators. In this Web Application, there is an integrated view of Telecare (e.g. movement, habits) and Telehealth (e.g. body weight, blood pressure) data offering also graphical and statistical facilities. Forwarded from the SPP alerts are presented real-time on screen by the Consumer Applications and, if this is the case, other relevant actions take place too, like SMS sending to relatives, doctors and/or operators.

inCASA is an EU co-funded pilot project with a combination of industry and academic partners and has already deployed its pre-mature solution to five European pilots (hospitals or social services). The primary measurable indicators during the pilots include the overall elderly patient satisfaction with the provided services, enhancement of their self-reliance and living conditions and the added value of this service model (i.e. reduced hospitalization of patients and/or response times to emergencies). First results are already collected and satisfy the doctors/operators aim for Telehealth-Telecare integration. One of their main targets that can be supported by the aforementioned integration is the early detection of health deterioration triggered by deviation in user’s habits. In our presentation, we will report on latest results and discuss challenges and benefits of the Telehealth and Telecare monitoring combination.

